# Association of American Medical Editors

**Published:** 1873-05-15

**Authors:** 


### ASSOCIATION OF AMERICAN MEDICAL EDITORS.

This organization held its annual meeting in the Polytechnic Institute, on Monday evening, May 5th, 1873. As has usually been the case, only a limited number of representatives of the Medical press were present. Dr. Theophilus Parvin, President of the Association, called the meeting to order. The Secretary read the minutes of the previous meeting. The President read an address on the relations between Medicine, Philosophy and Literature, which was listened to with interest by an appreciative audience. The address gave evidence of much literary research on the part of its author, and was written in elegant style. The thanks of the Association were tendered, and a copy of the address requested for publication. The Committee on Prize Essays made no report. The following officers were elected for the ensuing year: President, W. K. Bowling, of Nashville, Tenn.; Vice-President, W. S. Edgar, of St. Louis, Mo.; Secretary, F. H. Davis, of Chicago, Ill.

The next meeting is to be held in Detroit, Michigan, on Monday evening preceding the first Tuesday in June, 1874.

